# Living With the Fear of Recurrence in Benign Paroxysmal Positional Vertigo

**DOI:** 10.1111/hex.70551

**Published:** 2026-01-05

**Authors:** Seo‐Jin Lee, Yujin Hur

**Affiliations:** ^1^ Graduate School Dongguk University WISE Gyeongju Korea; ^2^ Department of Nursing Dongguk University WISE Gyeongju Korea

**Keywords:** dizziness, patient perspectives, phenomenology, qualitative research, self‐management, vertigo

## Abstract

**Background:**

Benign paroxysmal positional vertigo (BPPV) is a common vestibular disorder characterized by brief vertigo episodes. It often leads to functional limitations, psychological distress, and fear of recurrence.

**Objective:**

The purpose of this phenomenological study was to gain a deep understanding of the lived experiences of patients with BPPV.

**Methods:**

Participants were initially recruited through convenience sampling at a BPPV specialist clinic, with additional participants identified via snowball sampling. Data were gathered through individual, in‐depth interviews.

**Findings:**

A central theme, “Reconstructing meaning in the face of an unpredictable illness,” captured patients' transformative journey from shock and confusion to adaptation and renewed self‐awareness. Six themes emerged from data: “Shock and confusion from unexpected illness,” “Unbearable symptoms affecting daily life,” “Persistent anxiety about recurrence leading to emotional fatigue,” “Heightened health awareness following the illness experience,” “Disappointment with treatment support and efforts to seek alternatives independently,” and “Empathy from others and support from family as emotional anchors.”

**Conclusion:**

Patients with BPPV experienced fear and dread of recurrence, while simultaneously struggling to develop their own coping strategies to address unresolved questions and concerns. Recognizing this need can help identify areas requiring professional intervention. Although the symptoms and fears associated with BPPV may be underestimated due to the perceived mildness of the condition, the impact of BPPV on patients' overall well‐being is significant.

**Patient and Public Contribution:**

Patients and the public were not directly involved in the design or conduct of this study. However, the research was grounded in the lived experiences of individuals with BPPV, whose narratives were central to shaping the research questions and analysis. Their perspectives provided valuable insights into the physical, emotional, and social impacts of BPPV, ensuring that the findings reflect issues most relevant to those affected and guiding potential directions for future patient‐centered nursing.

## 1 Introduction

Vertigo or dizziness, is one of the most common symptoms reported by individuals [1]. It is defined as the sensation that either oneself or the surrounding environment is moving, even when no actual movement occurs [2]. Among the various conditions that can cause dizziness, benign paroxysmal positional vertigo (BPPV) was identified as the most prevalent diagnosis [1]. The exact cause of BPPV remains unknown [3], but it can be associated with factors, such as aging, head and neck trauma, ischemia, vestibular degeneration, and other ear disorders [4]. In 2024, the population of older adults in Korea, defined as those aged 65 years and older, was projected to be 9.93 million, accounting for 19.2% of the total population [5]. Because the proportion of older adults in Korea continues to grow, we expect that the incidence and prevalence of BPPV will also increase [1].

BPPV is a mechanical disorder of the peripheral vestibular system caused by dislodged otoconia, which are primarily composed of calcium carbonate crystals [6]. Symptoms are triggered by changes in head position relative to gravity [7]. Common symptoms include balance disturbances, fatigue, slow walking speed, an increased number of falls, and exercise intolerance [3, 8]. The term “paroxysmal” refers to the sudden onset of vertigo that occurs with any change in position [2]. In severe cases, vertigo could be triggered by any head movement, leading to persistent episodes that may be accompanied by nausea and vomiting [9]. Furthermore, these symptoms could last for months or years, or they may recur over several years [10].

Although BPPV is classified as benign, it significantly limits patients' daily activities [6, 11]. The term “benign” refers to the generally positive prognosis, indicating that BPPV is not caused by any serious central nervous system disorder. Approximately 20% of patients recovered spontaneously within 1 month, and up to 50% recovered within 3 months of follow‐up [12, 13]. While around 95% of BPPV patients underwent canalith repositioning therapy (CRT) [9], recurrence rates remained notable, with about 20% experiencing recurrence within 1 year and another 20% within 5 years [3]. If left untreated, BPPV increased the risk of falls and impaired daily living, with falls, depression, and impairments in daily living being more common among older patients [14, 15]. This not only added a burden on caregivers but also led to societal costs, such as reduced family productivity and a higher risk of nursing home admissions [2]. The complications associated with BPPV were varied, and the associated anxiety and depression often remained unaddressed [16]. The episodic nature of BPPV, characterized by sudden episodes of vertigo and dizziness, can be particularly anxiety—inducing for patients [17].

Previous studies on BPPV have primarily focused on quantitative research. Researchers have investigated several aspects of BPPV, including the increased medical costs associated with dizziness and vertigo [1], the relationship between migraine and BPPV, risk factors for BPPV recurrence [18], and various treatment options, such as medication and rehabilitation [6]. Important themes examined in research involving patients with BPPV include depression and anxiety [16], falls [19], and quality of life [8]. Most of these studies were systematic literature reviews or quantitative studies.

Analysis of existing research revealed that quantitative studies have primarily addressed the psychological issues, treatment, and management of patients with BPPV, while qualitative research has remained limited. In light of the lack of comprehensive understanding of patients with studies on BPPV, this study aimed to explore patients' disease experiences in depth, thereby offering a basis for effective nursing interventions and contributing to the development of nursing knowledge.

## 2 Methods

### 2.1 Design

This study employed Giorgi's descriptive phenomenology [20] to understand the meaning of patients' experiences with BPPV.

### 2.2 Study Setting and Recruitment

The participants in this study were individuals who visited an otolaryngology clinic and had previously experienced BPPV. The inclusion criteria required that participants be diagnosed with BPPV and able to accurately describe their experiences. We used convenience sampling to recruit participants from a BPPV clinic, and then employed a snowball technique based on recommendations from initial participants. In phenomenological research, data collection typically continues until saturation is reached, which means that no new insights are emerging from participants [21]. After discussions among the researchers, it was determined that sufficient saturation had been achieved, and therefore concluded interviews after collecting data from seven participants.

### 2.3 Data Collection

We collected data through one‐on‐one, in‐depth interviews conducted between February and July 2025. After the interviews, if participants had additional comments or required clarification, we conducted additional telephone interviews.

The interview questions were specifically designed to align with the content of the interviews (Table 1). Phenomenological conversations involve building on insights gathered during the discussion [22], so a trained researcher continued to ask relevant questions based on cues presented by the participants. During the interviews, participants shared their thoughts and feelings related to the topic by using the questionnaire, while their nonverbal cues were observed throughout. To minimize bias, the researchers pre‐described in advance what we believed patients with BPPV might experience and engaged in ongoing discussions to refine these assumptions during data collection. The researchers articulated our preconceived notions prior to the interviews, stating: “Patients may be fearful of BPPV symptoms,” and “Patients may have developed their own coping strategies for BPPV.” Efforts were made to avoid leading questions to reduce the influence of the researchers' preconceived ideas on the participants' experiences and responses.

**TABLE 1:** Semi‐structured interview guide.

Question
– Have you heard of or are you aware of the disease BPPV? – When were you diagnosed with BPPV? – What was your experience upon being diagnosed with BPPV? – How did you feel when you were diagnosed with BPPV? – What difficulties did you experience with BPPV? – What resources or methods did you use to overcome these difficulties? – What social and emotional changes have you experienced since being diagnosed with BPPV? – How has BPPV affected your life? – What concerns do you have for the future? – Is there anything else you would like to share?

Abbreviation: BPPV, benign paroxysmal positional vertigo.

### 2.4 Ethical Considerations

Before the data collection began, the Institutional Review Board at the researcher's institution reviewed and approved the study (no. DGU IRB 20250002). The purpose of the study was explained to them over the phone, and interviews were then scheduled for specific dates and times. During the interviews, participants were informed about the research procedures, including the recording of the sessions and the subsequent deletion of those recordings. They were also informed of their right to withdraw from the study at any time. We collected written informed consent from each participant. The researcher transcribed the recorded interviews and compared them with written notes. The recordings were anonymized using participant codes, and access to the transcriptions and consent forms was restricted to the researcher.

### 2.5 Data Analysis

The use of phenomenology as a research methodology effectively achieved the goal of gaining a deeper understanding of the experiences of living with BPPV. This approach aligned with the study's objectives as it allowed participants to focus on their individual lived experiences and describe how BPPV affects their daily lives. This method enabled us to capture human experiences, providing a more precise and meaningful understanding of participants' subjective perceptions.

In this study, we began by repeatedly reading the transcripts to understand the overall meaning of the situations described by the participants. After gaining a general sense of these meanings, we identified several semantic units from the participants' statements. We then reexamined the entire transcripts in detail, focusing on the participants' experiences with BPPV as a phenomenon, which allowed us to identify these semantic units. This process resulted in a total of 162 semantic units derived from the utterances of the seven participants. Next, through collaborative meetings, we transformed the semantic units into theoretically articulated units. We discussed these units and used creative transformations to integrate them into a comprehensive structure for the research phenomenon. As a result, we described the essential structure of the phenomenon, which led to the identification of six themes, 15 subthemes, and 48 semantic units.

### 2.6 Rigor and Reflexivity

This study ensured rigor based on Munhall's criteria [23] of resonance, reasonableness, representativeness, recognition, raising awareness, readability, relevance, revelation, and responsibility. To ensure resonance and reasonableness, we preserved the true meanings and emotions in participants' narratives and analyzed them carefully to establish logical connections with the research questions. Representativeness was achieved by including participants of diverse genders, ages, symptom durations, and recurrence experiences. For recognition and raising awareness, we illustrated how BPPV affects daily life, emotions, and social relationships, drawing attention to the often‐overlooked suffering and anxiety of patients. Readability was maintained by avoiding medical jargon and presenting interview excerpts clearly. Regarding relevance and revelation, the findings highlighted the need for nursing interventions and educational resources grounded in real patient experiences, offering new insights into the emotional dimensions of BPPV. Additionally, the research team has experience in qualitative research and regularly attends academic conferences to stay informed about current trends. During data analysis, we checked any unclear statements with participants to improve accuracy, and all data were kept confidential.

## 3 Findings

### 3.1 Characteristics of Participants

Participant characteristics are reported in Table 2.

**TABLE 2:** General characteristics (*N* = 7).

ID	Gender	Age	Currently employed	Marital status	Education level	Living with	Duration of illness
1	Male	41	Yes	Married	University	Spouse, 1 child	1 month
2	Female	44	Yes	Married	High school	Spouse, 1 child	1 month
3	Female	65	Yes	Married	High school	Spouse, 1 child	18 months
4	Female	75	No	Married	High school	Spouse	36 months
5	Female	43	No	Married	University	Spouse, 2 children	3 days
6	Female	42	Yes	Single	University	Lives alone	34 months
7	Female	35	Yes	Married	University	Spouse	2 months

### 3.2 Main Findings

We consistently engaged in discussions and inquiries to understand and interpret the significance of patients' experiences with BPPV. Therefore, the central phenomenological question of this study was, “What is the meaning of patients' experiences with BPPV?” The experience of living with BPPV reflected a transformative journey that began with the initial shock and disruption caused by sudden illness and progressed to a reconstruction of meaning through self‐awareness, independent coping strategies, and the supportive empathy of others. This central theme, “Reconstructing Meaning in the Face of Unpredictable Illness,” captured the essence of the participants' experiences. The identified themes and subthemes are presented below (Table 3).

**TABLE 3:** Subtheme and theme.

Subtheme	Theme
Fear of sudden and uncontrollable symptoms	Shock and confusion from unexpected illness
Pain and discomfort during the examination and treatment process	
Disoriented and overwhelmed by the symptoms	Unbearable symptoms affecting daily life
Avoidance of daily activities due to fear of symptoms	
Guilt and anxiety about burdening coworkers	
Living in constant anxiety about symptom recurrence	Persistent anxiety about recurrence leading to emotional fatigue
Staying tense, sensitive to minor bodily sensations	
Anticipation of symptom recurrence leading to emotional burnout and depression	
Increased attention to physical condition	Heightened health awareness following the illness experience
Efforts to improve lifestyle and manage symptoms	
Frustration with limited treatment options and the need to endure symptoms	Disappointment with treatment support and efforts to seek alternatives independently
Turning to folk remedies in search of relief	
Unsuccessful self‐management attempts based on social media information	
Emotional comfort through empathy from others with similar experiences	Empathy from others and support from family as emotional anchors
Family support as a source of psychological stability	

#### 3.2.1 Theme 1. Shock and Confusion From Unexpected Illness

Most participants described a profound sense of losing control over their bodies because of the sudden onset of physical symptoms in everyday situations. Symptoms, such as dizziness, vomiting, and loss of balance were not only uncomfortable but also disrupted and threatened their entire daily routines. The diagnostic process, which required repeated exposure to movements that triggered symptoms, was accompanied by both physical distress and emotional distress. From the moment symptoms began, many participants felt that their daily functioning was temporarily paralyzed, which led to ongoing anxiety and tension because of the unpredictability of when and where the symptoms might recur.

##### 3.2.1.1 Subtheme 1. Fear of Sudden and Uncontrollable Symptoms

Most participants described experiencing intense confusion and fear when symptoms, such as dizziness and nausea appeared suddenly and without warning, often causing the sensation that the world was spinning. These episodes went beyond discomfort and significantly interfered with daily life. Some participants reported feeling so incapacitated that they could not walk to the hospital without assistance. Resting or sleeping comfortably also became difficult, and even ordinary postural changes were perceived as threatening. Many participants reported feeling overwhelmed and helpless, as if they were in a situation beyond their ability to manage or understand.

“I was resting in bed and woke up to get up, but, um, suddenly, the ceiling in this room felt so spinning that I was like, ‘Oh, what's wrong?’ and I was going to lie down and rest. But the dizziness didn't stop, and the ceiling was spinning more severely…” (Participant 1). “I can't do anything with it. It's hard to lie down. I even stayed up all night sitting on a chair on the couch (laughs). I was so scared to the side that I couldn't lie down and I was dizzy (laughs).” (Participant 4).

##### 3.2.1.2 Subtheme 2. Pain and Discomfort During the Examination and Treatment Process

Many participants reported experiencing both physical distress and emotional discomfort during the BPPV diagnostic process. During the examination, when their heads were tilted in specific directions, they experienced intense dizziness, as though they were falling, and the pain was severe enough to cause them to scream. They also described the chaotic process of independently searching for information and visiting multiple hospitals to obtain a precise diagnosis and treatment.

“The examination was a little harder than I thought” (Participant 1). “Even during the examination, I was on the right side, but on the left… I felt like I was falling to the left even though I didn't lean. So I screamed” (Participant 4). “It was the weekend, and the emergency room didn't work. And the treatment… I was like, well, this isn't working properly, and now I'm back. I didn't know it was otolithiasis at the time” (Participant 5).

#### 3.2.2 Theme 2. Unbearable Symptoms Affecting Daily Life

Most participants described experiencing significant difficulties in their daily lives because of BPPV, which placed them in situations characterized by fear and uncertainty. They reported a loss of control over their bodies and a general decline in their ability to perform everyday tasks, such as childcare. The constant fear that even a single movement could trigger symptoms led them to avoid basic activities, including going outside or doing housework. These limitations also affected their work environments, where participants felt ongoing anxiety about the recurrence of symptoms, as well as a sense of burden and guilt regarding the possibility of negatively affecting colleagues. Their reduced ability to maintain daily routines and social roles reflected not only physical restrictions but also resulted in emotional withdrawal and a sense of isolation.

##### 3.2.2.1 Subtheme 1. Disoriented and Overwhelmed by the Symptoms

Many participants described feelings of helplessness caused by persistent dizziness that interfered with even basic head movements. They reported feeling powerless because they could not control their bodies. Simple actions, such as bending down or turning their heads, provoked dizziness, which made everyday activities, including standing or walking, extremely difficult.

“When I turned my head, I suddenly felt dizzy, which made it very difficult. I continued to experience dizziness for about 10 days. I was unable to do anything” (Participant 2). “It was very severe at that time. I could not stand up, I could not walk, I felt as though I would continue vomiting, I felt unwell, and I was unable to do anything” (Participant 5). “Therefore, I did not even dare to move at all. I managed to find a specific position in one direction where I was not dizzy, and I had to remain in that position” (Participant 6).

##### 3.2.2.2 Subtheme 2. Avoidance of Daily Activities Due to Fear of Symptoms

Most participants described avoiding normal daily activities because of uncertainty and fear regarding when and where their dizziness might recur. Even basic forms of mobility, such as going outside, driving, or climbing stairs, were avoided because of concerns that these actions could result in accidents. As a result, some participants reduced outdoor engagement and restricted interpersonal relationships. Over time, these participants limited both their choices and participation in everyday activities, which ultimately resulted in feelings of social withdrawal and diminished self‐worth.

“The thing I was most afraid of was going outside. So, what if I suddenly get dizzy while driving? What if I suddenly fall down while going up the stairs?” (Participant 1). “It's really… It starts with cooking, and there's nothing to do without caring about it” (Participant 2). “But I've been driving for about 30 years, but I quit driving and I'm scared to drive. I thought I should prevent it in advance, so I quit driving (laughs)” (Participant 4). “I can't go on rides after that. Especially, I can't ride swings or anything that I can barely ride, even swings. It's like a ride.” (Participant 7).

##### 3.2.2.3 Subtheme 3. Guilt and Anxiety About Burdening Coworkers

Several participants described experiencing ongoing anxiety and tension related to their work because of the possibility that sudden symptoms could occur in the workplace. Among these participants, awareness that their condition might burden coworkers resulted in persistent feelings of guilt.

“When I have symptoms in between, I feel sorry for my colleagues whom I work with, and it affects my work a little bit” (Participant 6). “Especially when I go to work now, when those symptoms come out, I think I can't work properly and it'll interfere with my work… As I continue to work, I feel anxious, and I wonder when this symptom will appear” (Participant 7).

#### 3.2.3 Theme 3. Persistent Anxiety About Recurrence Leading to Emotional Fatigue

Most participants described living in a state of ongoing tension, uncertain when or under what circumstances their symptoms might return. Past painful experiences led them to interpret even ordinary bodily sensations as early warning signs, which contributed to heightened vigilance and sustained anxiety. The possibility of recurrence caused them to be overly cautious in everyday life, and this prolonged state of worry gradually developed into emotional exhaustion and, in some cases, depression. These emotional reactions extended beyond immediate responses to physical symptoms and ultimately shaped how participants perceived and approached their overall daily lives.

##### 3.2.3.1 Subtheme 1. Living in Constant Anxiety About Symptom Recurrence

Most participants reported living with ongoing anxiety about the potential recurrence of dizziness. Many remained constantly alert, fearing that symptoms could occur without warning, whether upon waking, during daily activities, or while outdoors. This anxiety persisted even during periods without symptoms.

“I think my biggest concern is that I'm getting dizzy again. Well, there's always a little bit of that spark left in my body” (Participant 1). “Oh, I'm scared that I'm going to be dizzy. When I wake up in the morning, I first look at the ceiling. I'm living in that kind of fear” (Participant 3). When I checked the number of months, it was like in about five months? I got a recurrence. But now it's been more than five months, and I think it'll be okay (laughs), but I'm more anxious” (Participant 4). “If I suddenly experience this much dizziness when I'm outside, I'm completely helpless. I could really call 911 and go to the hospital, oh no. I think I've had this constant anxiety” (Participant 7).

##### 3.2.3.2 Subtheme 2. Staying Tense, Sensitive to Minor Bodily Sensations

Many participants reacted sensitively to mild dizziness and physical sensations they detected in daily life, recognizing these as precursors to symptoms and experiencing anxiety. Memories of previous severe symptoms triggered immediate alertness to even small changes in their current condition, leading them to constantly check their bodies and question whether the symptoms would recur.

“I don't know when or where the symptoms will come from, so I keep paying attention to my body's condition. Even if my body is a little weird, I wonder if it's a recurrence. I overreacted (Participant 1).” “Sometimes when I feel mildly dizzy like this, I suspect this is otolithiasis. It was too much at the time (Participant 7).”

##### 3.2.3.3 Subtheme 3. Anticipation of Symptom Recurrence Leading to Emotional Burnout and Depression

Many participants reported experiencing emotional exhaustion and depressive feelings because of ongoing anxiety about symptom recurrence and a sense of helplessness in managing everyday life. Their mood progressively declined during symptom episodes, and concerns about the long‐term effect of the condition expanded into feelings of loneliness and a significant psychological burden.

“I think I should keep in mind the impact this disease can have on my whole life. It's kind of scary and sometimes lonely” (Participant 1). “It's just… It's all just… How should I say this? I think it wasn't good at all. I've been… I've been… I've been feeling depressed…” (Participant 2). “I was a little depressed because I thought that the symptoms would be repeated” (Participant 6).

#### 3.2.4 Theme 4. Heightened Health Awareness Following the Illness Experience

To prevent the recurrence of symptoms, many participants attempted to better adapt to bodily changes and actively control their daily lives. They incorporated physical condition management into their routines by avoiding intense activities, ensuring sufficient rest, and maintaining regular exercise. In addition, these participants implemented specific strategies to reduce the likelihood of recurrence in areas they believed could be controlled. They did this by reflecting on and adjusting repetitive behaviors, such as posture, sleep patterns, and eating habits.

##### 3.2.4.1 Subtheme 1. Increased Attention to Physical Condition

Most participants became more attentive to their physical condition than they had been before their illness, in an effort to prevent symptom recurrence. These participants monitored signs of fatigue and subtle physical changes with heightened sensitivity. Recognizing a connection between their condition and the onset of symptoms, they consciously avoided overexertion and made deliberate efforts to maintain physical balance through rest and light exercise.

“I used to think it would be okay if there was something wrong with my body, but now I'm more attentive to the signals my body sends” (Participant 1). “I think I tend to have these symptoms when I'm not in good condition, so I exercise a little, and when I'm a little tired, I reduce my activities and take a rest. I tried that kind of method first” (Participant 6).

##### 3.2.4.2 Subtheme 2. Efforts to Improve Lifestyle and Manage Symptoms

Most participants made conscious efforts to adjust their daily postures and movements to prevent the recurrence of symptoms. These changes were especially evident in repetitive behaviors, such as sleep posture and movements upon waking. They also engaged in ongoing self‐management strategies by reviewing existing habits and modifying aspects of their lifestyle to reduce the likelihood of symptom recurrence.

“As soon as I woke up, I stood up and turned my head or moved my body naturally without thinking, but now even if I do this, I think I'm getting used to it slowly and carefully.” (Participant 1). “If I lie on one side, I tend to use the upright position because I'm afraid I'll get symptoms. If I put my pillow high and lean my head a little bit, I heard it can be prevented, so I try a lot.” (Participant 3). “Since my body is weak now, I need to work out more physically by paying more attention to my body. I need to eat well and exercise well so that I can protect my body” (Participant 5).

#### 3.2.5 Theme 5. Disappointment With Treatment Support and Efforts to Seek Alternatives Independently

Many participants expressed disappointment and frustration with the lack of clear and effective treatment options, which led them to manage their symptoms independently rather than relying solely on medical interventions. They used folk remedies, online information, and self‐directed strategies, such as adjusting posture, taking health supplements, and engaging in self‐treatment. However, these methods were often only temporarily or minimally effective and, in some cases, resulted in physical discomfort or increased confusion about their condition.

##### 3.2.5.1 Subtheme 1. Frustration With Limited Treatment Options and the Need to Endure Symptoms

Many participants reported frustration because there were no definitive treatment solutions, even after they underwent multiple tests. They described both the emotional and physical burden of managing symptoms independently without meaningful medical relief. In many cases, incomplete recovery after treatment resulted in ongoing discomfort, which led to repeated visits to medical facilities or, at times, hospitalization to seek better symptom management.

“I went to two or three clinics because I was so dizzy at the time, but they said they didn't have any medicine. Just a test… Just a test and no medicine… It's… It's not like I can do a procedure, and my dizziness… It's a little hard for me to endure it” (Participant 2). “I guess it didn't work out at once, so the symptoms didn't go away completely. I desperately wanted to be admitted to the hospital. My condition was so bad” (Participant 5).

##### 3.2.5.2 Subtheme 2. Turning to Folk Remedies in Search of Relief

Several participants, recognizing the lack of clear medical treatments, repeatedly engaged in self‐directed attempts to relieve their symptoms, such as changing their posture or head position. In addition, they purchased and consumed health supplements found online, viewing these efforts as potential ways to ease discomfort or discover clues toward recovery.

“I really wanted to get better. I ate well and drank plenty of water. I don't think there was anything else” (Participant 2). “Someone posted something about otolithiasis on the Internet, so I bought it, and I ate a little…” (Participant 3). “As the recurrence was repeated, I followed folk remedies on social media at home and did not go to the hospital” (Participant 4).

##### 3.2.5.3 Subtheme 3. Unsuccessful Self‐Management Attempts Based on Social Media Information

Several participants attempted to manage their symptoms independently by relying on information and video content obtained through social media and online platforms. Although these resources initially provided a sense of hope, these attempts rarely resulted in meaningful improvement and often caused physical discomfort.

“Even if I watch YouTube and copy it, it doesn't work. Repositioning isn't as good as I thought, and nothing gets better quickly. It only causes vomiting” (Participant 6). “I copied what I watched on YouTube, but it didn't work” (Participant 7).

#### 3.2.6 Theme 6. Empathy From Others and Support From Family as Emotional Anchors

Many participants were able to ease their loneliness and anxiety through connections with individuals who understood and empathized with their suffering. Hearing from others who had experienced similar symptoms helped them realize that they were not alone, which played a key role in alleviating feelings of isolation. Consistent emotional support and physical care from family members were described as essential in helping participants endure the challenges of their symptoms. The presence of someone nearby provided a sense of psychological security and comfort during difficult times.

##### 3.2.6.1 Subtheme 1. Emotional Comfort Through Empathy From Others With Similar Experiences

Many participants experienced emotional relief when they heard the stories of others who had faced similar struggles, particularly during periods of loneliness when they felt that no one could fully understand their condition. Reading online posts or learning about cases among acquaintances confirmed that others also shared the specificity and severity of their symptoms. This recognition enabled participants to feel seen, understood, and emotionally supported.

“At first, I just went in to look for information, but when I saw someone post that it was the hardest thing not to understand by anyone, I really sympathized with this. I am not alone. It was somewhat comforting to find people who understood that this symptom was difficult to explain by simply saying it was dizzy” (Participant 1).

“I looked at the Internet, listened to the stories of people around me… We depend on each other and share it” (Participant 3).

##### 3.2.6.2 Subtheme 2. Family Support as a Source of Psychological Stability

Many participants found significant comfort in the emotional and physical support provided by their family members, particularly during periods when symptoms were severe. The warm encouragement and attentive care of spouses played a vital role in calming participants' anxiety and helping them endure difficult moments. The presence of a supportive family member offered reassurance, and the sense of not being alone contributed greatly to participants' psychological stability.

“The person who helped me the most emotionally was my wife. She was always around me saying, “It's okay now. It'll get better soon.” And that continued support… seemed to be a big driving force that allowed me to endure” (Participant 1). “My husband has helped me a lot now. I felt I needed someone next to me then” (Participant 4). “When the symptoms suddenly worsen, I get dizzy enough not to be able to move, so if someone is next to me, I feel a little comfortable emotionally” (Participant 6).

## 4 Discussion

This study explored the meaning and essence of patients' experiences with BPPV using a descriptive phenomenological approach in their daily lives because of the sudden onset, unpredictability, and recurrence of their symptoms. These disruptions resulted in physical, emotional, and social distress. As participants managed limited treatment options and attempted to address their symptoms independently, emotional support from family members and empathy from others with similar experiences became essential sources of psychological stability.

Most participants experienced significant confusion and fear because of the sudden onset of BPPV symptoms, which appeared without warning. Patients with BPPV experienced brief episodes of spontaneous positional nystagmus, accompanied by a sudden sensation of spinning during everyday movements, such as turning or tilting the head forward or backward. These symptoms made it challenging to maintain balance and compromised individuals' stability [24]. The unexpected changes provoked intense fear and terror in patients [17]. Many participants in this study also visited a hospital because of these symptoms. However, they reported severe discomfort and pain because of the intense dizziness induced during the BPPV diagnostic tests. The Dix–Hallpike and Supine Roll tests, which are crucial for diagnosing BPPV, could trigger a re‐experience of symptoms for patients [2].

Participants reported that their experiences with BPPV extended beyond physical symptoms; the condition significantly affected their daily lives, emotions, and social relationships. Many participants avoided going out because of their symptoms and experienced functional limitations in childcare and everyday activities. This finding is consistent with previous research, which showed that vertigo can negatively affect social interactions, work, and overall quality of life, including physical, functional, and emotional aspects [11, 25]. We found that the experience of symptoms in patients with BPPV was not limited to physical issues; instead, the broader changes in their lives and the emotional responses resulting from these changes must be addressed. Therefore, comprehensive nursing interventions should consider not only physical symptoms, but also changes in patients' daily functions, family dynamics, and social relationships.

Many participants were well aware that BPPV is a disease with a high risk of recurrence because of its nature, and therefore experienced constant vigilance and anxiety in their daily lives. The distressing nature of their symptoms resulted in heightened sensitivity to even minor bodily changes, while concerns about recurrence often led to feelings of depression. Although the term “benign” typically suggests a harmless condition, it is clear that the burden faced by patients with BPPV involves significant challenges [6]. Patients with recurrent BPPV were shown to have higher levels of trait anxiety compared to patients experiencing their first episode [26]. Additionally, those in the recurrence group tended to show increased levels of state anxiety. This finding aligns with participants' reports of the unpredictability and constant tension related to recurrence in this study, indicating that repeated symptoms can increase anxiety and lead to emotional exhaustion. Because of the recurring nature of BPPV, long‐term nursing interventions are crucial which should focus on educating patients about coping strategies for recurrence and promoting emotional stability.

Although the exact cause of BPPV remains unclear, most participants showed a commitment to maintaining a healthy lifestyle, including improved sleep and posture. These behaviors can be considered positive coping mechanisms, reflecting a willingness to manage one's health despite concerns about symptom recurrence. Recently, bone health issues, such as reduced bone density and vitamin D deficiency, were reported to contribute to BPPV [27, 28]. Therefore, in addition to lifestyle modifications, comprehensive evaluation and management of vitamin D levels, nutritional intake, and osteoporosis are crucial.

Many participants who experienced recurrent episodes of BPPV turned to social media platforms, such as YouTube for support. Searching for BPPV on YouTube revealed numerous videos that offered tips for self‐treatment. BPPV is caused by dislodged otoliths entering the semicircular canals, which creates a false sense of rotation. The canalith repositioning maneuver (CRM) was widely recognized as an effective treatment method [29]. However, the specific type of CRM required depended on which canal contained the dislodged otoliths, making accurate identification of the affected canal crucial for effective treatment [30]. This reliance on social media suggested that these participants had not found sufficient information or trustworthy sources about their condition. Because they coped with fear and anxiety regarding recurrence, patients often tried to devise their own strategies to answer unresolved questions related to BPPV. This shows the need for professional intervention, particularly in the form of patient education, psychosocial support, and guidance on appropriate management strategies provided by healthcare professionals. Furthermore, although BPPV is often viewed as a non‐life‐threatening condition, the current findings indicate that it can significantly affect daily functioning and quality of life. Therefore, active involvement from healthcare professionals and the development of systematic nursing interventions are important.

Many participants reported that psychological stability was significantly supported by family, through emotional care and physical assistance provided by their spouses. Additionally, some participants reported that online communities helped reduce feelings of isolation and offered comfort. A qualitative study of patients with unilateral vestibular hypofunction, a condition similar to BPPV, also showed similar results [31]. These results indicate that social support is an important psychological resource for reducing the emotional isolation and anxiety experienced by patients with BPPV.

In Korea, BPPV is commonly referred to as “I‐seok‐jeung,” which literally means “a problem of stones moving inside the ear.” This terminology may contribute to a tendency to trivialize the condition. Some participants in this study expressed sentiments such as, “Since it is an invisible illness, it is difficult for others to understand,” which highlights the challenges in receiving empathy from others. These perceptions can overshadow the complex suffering and functional limitations associated with the condition, and may worsen patients' sense of psychological isolation. Therefore, in nursing practice, it is essential to honor patients' lived experiences while fully acknowledging and empathizing with the seriousness of their symptoms and the effect on their quality of life.

### 4.1 Strengths and Limitations of the Work and Recommendations for Further Research

This study has few limitations. Given the qualitative design, we focused on the subjective experiences of individual patients rather than conducting a systematic analysis of clinical characteristics, such as otolith location, frequency of attacks, or the presence of comorbidities. Therefore, the results should be interpreted with caution. In addition, this study is limited by the lack of patient and public contribution to the study design and conduct, which may have restricted the integration of patient and public perspectives in shaping the research process and interpretation of the findings. Despite these limitations, this study offered a detailed explanation of the lived experiences of patients with BPPV, contributing to a deeper understanding of their cognitive and emotional responses, changes in daily life, self‐management strategies, and information‐seeking behaviors. In particular, by using a descriptive phenomenological approach, we explored patients' internal distress and coping experiences. Furthermore, the significance of this study lies in providing a foundation for developing nursing interventions and designing educational materials that address the specific needs of patients with BPPV.

### 4.2 Practice Implications

Nurses should approach the care of patients with BPPV from a biopsychosocial perspective, recognizing that this condition impacts not only physical function but also psychological well‐being and social participation. A comprehensive understanding of BPPV must consider emotional distress, fear of recurrence, stigma, and the effects on daily activities and interpersonal relationships. Ongoing professional education about BPPV and their psychosocial consequences can enhance nurses' ability to provide effective, patient‐centered care. It is important to address BPPV‐related concerns with sensitivity and respect, ensuring that patients feel heard and supported. Nurses play a crucial role in guiding patients toward appropriate management strategies, fostering adaptation, and promoting overall quality of life.

## 5 Conclusion

This study explored the experiences of individuals living with BPPV, revealing the emotional distress, lifestyle limitations, and challenges in self‐management due to recurring and unpredictable symptoms. Although BPPV is not a life‐threatening condition, this study revealed that its recurring and unpredictable symptoms significantly influenced individuals' daily functioning, emotional stability, and social role performance, which ultimately contributed to a reduced quality of life. Participants frequently reported frustration with the limited or unclear medical treatment options, which led them to seek relief through folk remedies, social media, and anecdotal information from the Internet. While emotional support from empathetic peers and family members provided some psychological stability, the findings show that healthcare professionals need to offer not only physical treatment but also accessible education, reassurance, and structured support. Future research should develop comprehensive intervention strategies that address both the physical and psychological challenges of living with BPPV and decrease patients' reliance on unverified sources.

## Author Contributions

**Yujin Hur:** conceptualization, methodology, project administration, data curation, formal analysis, investigation, validation, writing – original draft, writing – review and editing, supervision. **Seojin Lee:** data curation, formal analysis, investigation, validation, writing – original draft, writing – review and editing.

## Funding

The authors received no specific funding for this work.

## Ethics Statement

This study was approved by the Institutional Review Board of Dongguk University (DG IRB 20250002).

## Conflicts of Interest

The authors declare no conflicts of interest.

## Data Availability

Due to the sensitive nature of the qualitative interview data, the datasets are not publicly available. Anonymized excerpts are presented in the article, and additional information may be requested from the corresponding author.
